# Antinuclear antibodies may predict the development of immune-related adverse events in asymptomatic patients treated with immune checkpoint inhibitors: results from a single-center cohort

**DOI:** 10.1007/s10238-024-01317-z

**Published:** 2024-04-10

**Authors:** Fulvia Ceccarelli, Francesco Natalucci, Licia Picciariello, Alessio Cirillo, Giulio Olivieri, Margherita Veroli, Simona Pisegna, Claudia Ciancarella, Alain Gelibter, Vincenzo Picone, Daniele Santini, Andrea Botticelli, Fabrizio Conti

**Affiliations:** 1 Department of Clinical Internal, Anesthesiological and Cardiovascular Sciences, Arthritis Center, Rheumatology, Sapienza University of Rome, Rome, Italy; 2 Department of Radiological, Oncological and Pathological Science, Sapienza University of Rome, Rome, Italy; 3 Research Unit of Clinical Immunology and Vaccinology, Bambino Gesù Children’s Hospital, IRCCS, Rome, Italy; 4 PhD Program in Immunology, Molecular Medicine and Applied Biotechnology, University of Rome Tor Vergata, Rome, Italy; 5 Division of Medical Oncology B, Policlinico Umberto I, Sapienza University of Rome, Rome, Italy

**Keywords:** Immune-checkpoints inhibitors, Anti-nuclear antibodies, Adverse events, Biomarkers

## Abstract

We aim at investigating the association between subclinical autoimmunity and immune-related adverse events (irAEs) in a cohort of patients treated by immune checkpoint inhibitors for solid metastatic cancer. In the context of an oncology/rheumatology outpatient clinic, we evaluated patients treated with anti-PD-1 or anti-PD-L1. Before treatment, each patient underwent a physical evaluation and a blood sample to identify the presence of a set of autoantibodies. Indeed, all the patients were followed during treatment to identify irAEs and to assess the association with autoantibodies. Fifty-one patients (M/F 16/35; median age 70 years, IQR 16.5) were evaluated; 34.8% of patients showed ANA positivity, 6.5% ENA positivity (anti-SSA), 4.3% Ratest positivity, and 2.1% (one patient) ACPA positivity. During a median period of 21 months (IQR 38.75), 39.2% of patients developed irAEs. Musculo-skeletal manifestations, in particular arthritis, were the most frequent. We found a significant association between the positivity for ANA and the development of irAES (*p* = 0.03, RR 2.01, 95% CI 1.03–3.92). Furthermore, the progression-free survival was significantly longer in patients developing irAEs compared to those who are not experiencing these events (*p* = 0.007). This study underlines the potential role of ANA positivity as a predictive biomarker for the development of irAEs.

**Supplementary Information:**

The online version contains supplementary material available at 10.1007/s10238-024-01317-z.

## Introduction

In the past decade, immune checkpoint inhibitors (ICIs) have emerged as the predominant therapeutic approach in the field of oncology, leading to a major shift in the cancer management, with a significant impact on patients prognosis. The increasing use of ICIs in oncological clinical practice focused attention on ICIs-related side effects, the so-called immune-related adverse events (irAEs). These last encompass a spectrum ranging from benign, self-resolving conditions to severe, life-threatening clinical presentations, potentially affecting every organ/system, including musculoskeletal system [1, 2].

When evaluated exclusively from an oncological perspective, irAEs could have been inadequately assessed and then underdiagnosed. Indeed, the application of a multidisci-plinary approach could provide more detailed information in terms of prevalence, phenotype, severity and management of irAEs. It is relevant that this shared approach plays an important role in the decision whether or not to continue ICIs treatment when these adverse events develop [3].

Taken all together, these observations opened new research fields focusing on the identification of biomarkers able to predict the development of irAEs and maximize the risk–benefit ratio of ICIs treatment [4].

If one can expect a relapse of a pre-existing autoimmune disease during ICIs administration [5], little is known about the evolution of the so-called “subclinical autoimmunity.” This condition, consisting of seropositivity for autoantibodies without any clinical signs or symptoms of autoimmune diseases, may be triggered by ICIs treatment, which would therefore act as a “second hit,” thus leading to the development of irAEs.

Building on these premises, the primary objective of our study was to investigate the association between subclinical autoimmunity and irAEs development in a cohort of patients treated by ICIs for solid metastatic cancer.

## Materials and methods

We evaluated a cohort of patients diagnosed with solid metastatic cancer as a part of an oncology/rheumatology outpatient clinic. In detail, the patients were referred from the Oncology Department, Policlinico Umberto I, Sapienza University of Rome, to the Arthritis Center of Sapienza University of Rome.

For the present analysis, we included all the patients eligible for treatment with ICIs targeting PD-1 (pembrolizumab, nivolumab) or PD-L1 (cemiplimab, avelumab, durvalumab). Patients with previous autoimmune diseases were excluded from the study.

Before starting immunotherapy, each patient underwent a complete physical examination. Furthermore, a blood sample was collected to identify the presence of specific autoantibodies before treatment starting. This evaluation included:

- Antinuclear antibodies (ANA) and anti-dsDNA, detected by means of indirect immunofluorescence [6, 7];
- Rheumatoid factor (RF), anti-citrullinated protein antibodies (ACPA), and extractable nuclear antigen antibodies (ENA) detected by using commercial ELISA kits (results evaluated according to the manufacturers’ instructions).

The study was conducted in compliance with the principles outlined in the Declaration of Helsinki, and informed consent was obtained from each patient.

### Follow-up

Patients were educated about possible irAEs and were explained how to recognize them. According to oncological diagnosis, disease stage, and medication, each patient was clinically followed and each side effect related to ICIs treatment as well as chemotherapy was reported.

Furthermore, data about the overall survival (OS) and the progression-free survival (PFS) were registered. A rheumatological evaluation was performed each time a suspicious sign or symptom emerged.

### Statistical analysis

The statistical analyses were performed using version 9.0 of the GraphPad statistical package. Normally distributed variables were summarized using the mean ± standard deviation (SD), and non-normally distributed variables by the median and interquartile range (IQR). Frequencies were expressed by percentage. Univariate comparisons between nominal variables were calculated using the chi-square test or Fisher’s exact test, where appropriate. Two-tailed p values were reported; p values less than 0.05 were considered significant. In the follow-up analysis, the estimated risk of development of a specific event was determined with the relative risk (RR).

## Results

We enrolled 51 patients (M/F 16/35; median age 70 years, IQR 16.5). Nineteen patients (41.3%) suffered from NSCLC, 15 (32.6%) from head and neck cancer, 5 (10.8%) from melanoma, 4 (8.7%) from urothelial, 6 (13%) from kidney, 1 (1.9%) from squamous cell carcinoma and 1 (1.9%) from Merkel cell carcinoma. Moving on treatment, 28 (54.9%) patients were treated with pembrolizumab, 19 (37.2%) with nivolumab, 2 (3.9%) with cemiplimab, 1 (1.9%) with avelumab and 1 (1.9%) with durvalumab.

A complete serological evaluation was available for 46 patients (90.2%). Sixteen patients (34.8%) were ANA positive with a titer of at least 1:80; specifically, according to ICAP classification, 13 were characterized by a homogeneous pattern (AC-1), 1 by centromeric pattern (AC-3), 1 by speckled pattern (AC-2), and 1 by both homogenous and speckled (AC-1 + AC-2). Moving to the ANA titer, 7 patients had an ANA title —1:80; 4— patients  1:160; 4 patients 1:320; 1 patient 1:640.

**Fig. 1:**
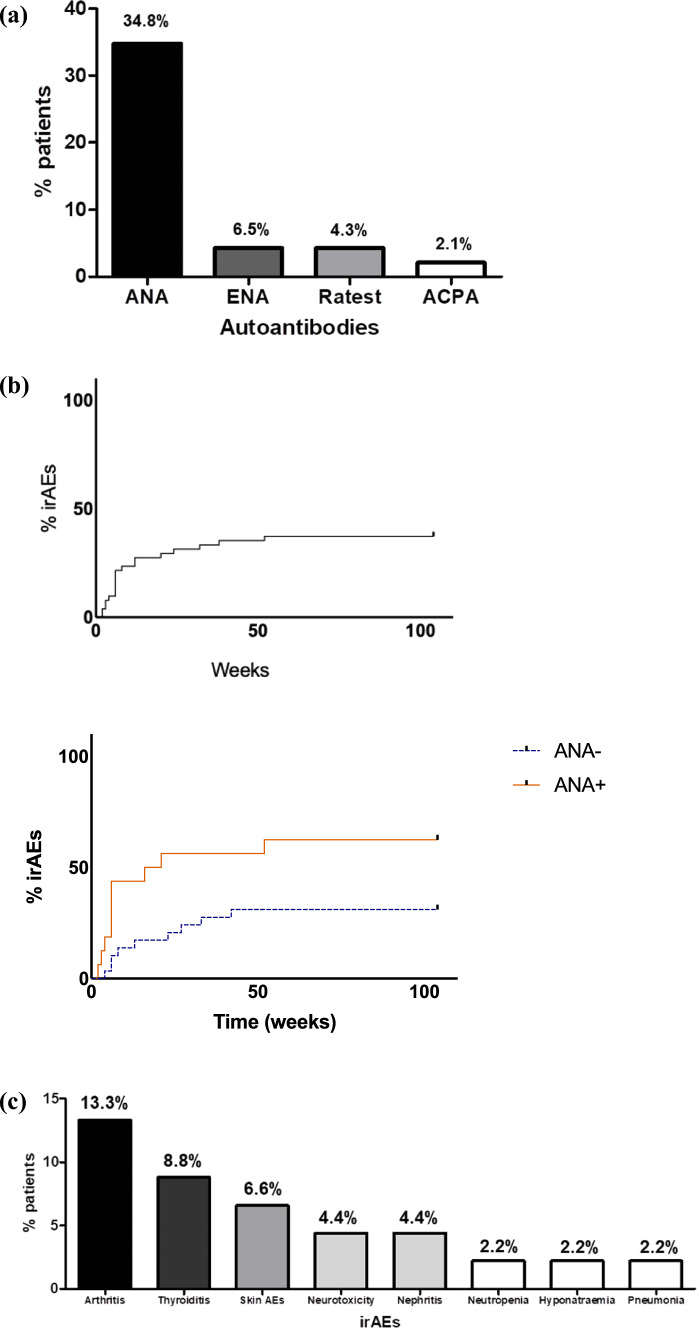
**a** Prevalence of autoantibodies in the analyzed cohort before immune-checkpoint inhibitors treatment, **b** Kaplan–Meier analysis of the occurrence of immune-related Adverse Events in the overall patients and by dividing ANA + and ANA − patients (Log-rank Mantel-cox test, (62.5% versus 31.0%; *p* = 0.029). **c** Prevalence of immune-related Adverse Events (irAEs) developed during follow-up

Furthermore, three (6.5%) were ENA positive and all of them showed a high titer positivity for anti-SSA antibodies; two were (4.3%) Ratest positive (one for IgM RF, IgA RF) and one (2.1%) showed ACPA positivity (Fig. 1a).

### Longitudinal analysis

Patients were followed up for a median period of 21 months (IQR 38.75). The median overall survival (OS) was 18 months (IQR 18) and the median progression-free survival (PFS) was 6 months (IQR 11).

During this period, 19 patients (41.3%) developed irAEs after a median interval equal 6 weeks (IQR 16; mean 13.05 weeks, SD 14.0). The Kaplan–Meier analysis is reported in Fig. 1b.

In detail, 6 patients (13.3%) experienced arthritis, 4 patients (8.8%) thyroiditis, 3 (6.6%) dermatological reactions, 2 (4.4%) neurotoxicity, 2 patients (4.4%) nephritis, 1 patient (2.2%) severe neutropenia, and 1 (2.2%) pneumonia (Fig. 1c). During observation, any adverse effects not directly attributable to immunotherapy were recorded but not considered as ICIs induced, including nausea, fatigue, low-grade anemia, peripheral neuropathic damage, hearing loss, and severe hyponatremia.

No differences were found in the irAEs prevalence according to administered drug.

### Analysis stratified for ANA positivity

**Fig. 2:**
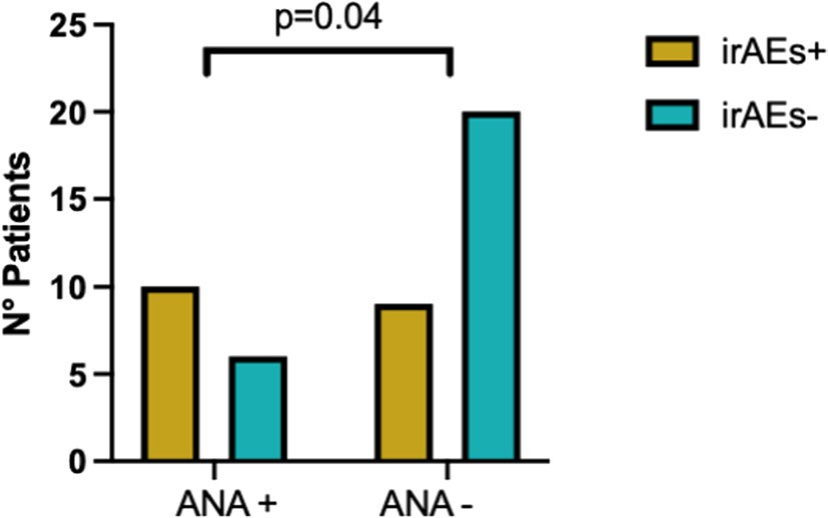
Incidence of immune-related Adverse Events (irAEs) in ANA + and ANA −  patients

Considering the high prevalence of ANA antibodies, we performed a comparison between ANA + and ANA − patients. Indeed, the incidence of irAEs was significantly higher in ANA + patients [10/16 patients (62.5%) versus 9/30 (31%), *p* = 0.03; Fig. 2]; furthermore, the relative risk (RR) of developing any irAEs for ANA-positive patients was 2.01 (95% CI 1.03–3.92; *p* = 0.04). Indeed, we stratified patients according to ANA titer: seven patients (43.7%) showed 1:80 titer, the remaining 9 showed a titer of ≥ 1:160. No differences were found according to ANA titer in the occurrence of irAEs (*p* = 0.14).

No differences were found between ANA + and ANA −  patients in terms of treatment response and PD-L1 expression (Supplementary Table 1-S1). Similarly, no differences were identified when comparing ANA + and ANA −  patients in terms of PFS [9.5 months (IQR 19) versus 5 months (IQR 9.5); *p* = 0.08) and OS [18.5 months (13.75) versus 18 months (IQR 20); *p* = 0.39]. When stratifying patients by the occurrence of irAEs, the PFS was found to be significantly longer in patients developing adverse events compared to those who did not experience these events [10 months (IQR 16.5) versus 4 months (IQR 9.5); *p* = 0.007].

Indeed, arthritis was found to be the most frequent "irAEs" and developed in six patients (30% of total irAEs). Three of them were treated with nivolumab and three with pembrolizumab. The serological features of these six patients are reported in Table 1: of note, 5 out of 6 (83.3%) were ANA positive, associated with other autoantibodies positivity in 4 patients. Of note, one patient positive for ANA, Ratest, and anti-SSA, developed lower limb purpura, xerostomia and lymphopenia associated with arthritis. Accordingly, a diagnosis of Sjögren Syndrome (SS) was made (patient 2).

**Table 1:** Serological status and treatment of six patients experiencing arthritis during treatment with immune-checkpoints inhibitors (ICIs)

Pt	Sex	Age	Time to onset (Weeks)	ANA	RATest	FR IgM	ENA	aSSA	aSSB	ACPA	Treatment	Evolution
1	M	71	4	1:80 (Ho)	0	0	1	250	0	0	PDN 10 mg/die	Exitus
2	F	82	6	1:160 (Ho)	0	32.7	1	274	0	0	PDN 5 mg/die + HCQ 200 mg/die	SdS diagnosis
3	M	75	2	1:80 (Ho)	81 IgM	63	0		0	0	PDN 10 mg /die	Persistent remission of arthritis after stopping GCs
4	F	74	2	1:160 (Ho)	0	0	0	0	0	0	PDN 10 mg/die + HCQ 200 mg/die	Introduction of HCQ due to persistence of arthralgias
5	M	60	8	0	0	0	0	0	0	0	PDN 25 mg/die	Lost to Follow-up
6	F	60	52	1:80 (Ho)	0	0	0	0	0	0	PDN 10 mg/die and + MTX 10 mg/weekly	Introduction of MTX due to persistence of arthritis

*Ho* homogenous, *PDN* prednisone, *SdS* Sjögren Syndrome, *GCs* glucocorticoids, *HCQ* hydroxychloroquine, *MTX* methotrexate

All the patients were treated with a median glucocorticoids (GCs) dose of 10 mg (IQR 5) with fast tapering. Treatment with cDMARDs was added in three patients; specifically, hydroxychloroquine (HCQ) was added in the patient diagnosed with SS and in a second patient due to the persistence of arthralgia at GCs tapering (patient 4). Finally, one patient received methotrexate (10 mg weekly) due to persistent arthritis despite GCs treatment; this patient was diagnosed with undifferentiated arthritis (patient 6).

## Discussion

In this single-center prospective study, we evaluated the association between a pre-existing condition of subclinical autoimmunity and the development of irAEs in patients treated by ICIs. Indeed, our investigation underlines the potential role of positivity for ANA as a predictive biomarker for the development of these immune-related side effects. This finding could contribute to a growing body of evidence highlighting the intricate relationship between autoimmunity and irAEs occurrence. In the last years, several studies have reported data about the development of immune-related conditions in patients treated by ICIs [3]. However, in the majority of cases, no data are available about the autoantibodies status before starting the treatment. Thus, little is known about the role of pre-existing autoimmunity, and the available data reported contrasting results. We previously described the case of a 69-year-old male patient affected by advanced cutaneous squamous cell carcinoma in the head and neck with pre-existing ANA positivity, developing arthritis 6 weeks after the introduction of nivolumab treatment [8].

A recent paper published by Hsu and colleagues, including ninety-three liver cancer patients treated with anti-PD1, suggested the role of ANA positivity in predicting the development and severity of irAEs. Furthermore, in this cohort, the time to develop irAEs was significantly shorter in seropositive patients [9]. Similar results were reported in a second larger cohort of NSCLC patients treated with nivolumab or pembrolizumab. Indeed, positivity for ANA, rheumatoid factor and anti-thyroid antibodies was an independent factor for the development of irAEs [10].

On the other side, other studies, prevalently evaluating cohorts of patients with NSCLC, did not find any predictive role for pre-existing ANA positivity in the development of irAEs [11–13].

These contrasting results could be related to different aspects. First, these studies are not comparable, due to the great heterogeneity of cancer types, ICIs therapy protocol, disease stage, comorbidities, and irAEs assessment. Not least, the cut off for autoantibodies positivity, especially ANA, was different in the published studies [10, 13].

Besides the prediction of the irAEs itself, Campochiaro and colleagues showed that the autoantibodies may predict the disease severity and the need for more aggressive therapy. However, in this study, the serological evaluation was contextual to the development of the adverse event and no information was available about autoantibodies before immunotherapy [14].

In light of these divergent findings, it is worth mentioning the first EULAR recommendations released in 2021 [15]. These guidelines advise against testing asymptomatic patients for autoantibodies, aiming to underscore that the decision to initiate ICIs therapy should not be hindered solely by the presence of a pre-existing autoimmune disease or sieropositivity for autoantibodies in asymptomatic patients. Consequently, our results should be interpreted in accordance with these recommendations, serving as a valuable contribution to the understanding of the relationship between autoimmunity and the likelihood of developing irAEs. Furthermore, in line with the same recommendations, irAEs could be viewed as potential markers of treatment efficacy. This is clearly reported by different studies, showing an improved PFS in ANA-positive NSCLC patients treated with anti-PD1 drugs [10, 16].

In our cohort, we did not find the association between ANA positivity and better survival. Nevertheless, this analysis was not the main objective of our study and the results could be influenced by cancer types, treatment protocol before ICIs treatment and disease stages.

Our study has some strengths and some limits. First, this is a longitudinal study performed in a dedicated oncological/rheumatological outpatient clinic with easy dedicated access for cancer patients. We thus evaluated each patient with any suspicious ICIs side effects, allowing us to early identify and treat rheumatological irAEs. Furthermore, we analyzed a homogenous cohort in terms of ICIs treatment, because all the patients were treated by anti-PD-1 or anti-PD-L1 drugs. The major limits were certainly the cohort size and the heterogeneity in cancer disease and stage. Indeed, various diagnoses could negatively influence the statistical power of survival analysis. Indeed. the statistical result needs to be confirmed on larger prospective studies. Moreover, the heterogeneity of oncological diagnosis and stage should be kept in consideration when evaluating our results. Finally, it could be interesting to evaluate the evolution of ANA-negative patients who became ANA-positive during ICIs treatment, but this was not the purpose of the present study and we do not have information about change in the serological status.

In conclusion, our results underline the potential role of ANA positivity as a predictive factor for irAEs development in oncological patients undergoing ICIs therapy.

## Supplementary Information

Below is the link to the electronic supplementary material.

Supplementary file1 (DOCX 13 kb)

## Data Availability

All data are reported in the text.
