# Supplementary material for: Antinuclear antibodies may predict the development of immune-related adverse events in asymptomatic patients treated with immune checkpoint inhibitors: results from a single-center cohort
Source: Clin Exp Med. 2024 Apr 10;24(1):72. doi: 10.1007/s10238-024-01317-z (PMC11006777; doi:10.1007/s10238-024-01317-z)
Supplement: Supplementary file 1 — Supplementary file1 (DOCX 13 kb) [file 10238_2024_1317_MOESM1_ESM.docx]

**Supplementary Materials**

**Table S1.** Clinical and laboratory features of ANA+ and ANA - patients.

|  | **ANA+** | **ANA -** | **p** |
| --- | --- | --- | --- |
| **N°** | 16 | 29 | - |
| **Age (Median, IQR)** | 71.7 (16.3) | 71.5 (15) | 0.90 |
| **Best Response** | | | |
| **Progression Disease** | 4 (25) | 12 (40) | 0.34 |
| **Stable Disease** | 5 (31.2) | 8 (26.6) | 0.99 |
| **Stable/Partial Response** | 4 (24) | 4 (13.3) | 0.42 |
| **Complete Response** | 0 (0) | 2 (6.6) | 0.53 |
| **Non evaluable** | 0 (0) | 1 (3.3) | 0.99 |
| **PD-L1 (TPS/CPS)** | | | |
| **Non-Measured** | 8 (50) | 12 (40) | 0.75 |
| **<1%** | 2 (12.5) | 4 (13.3) | 0.99 |
| **1-49%** | 3 (18.75) | 8 (26.6) | 0.72 |
| **>50%** | 3 (18.75) | 6 (20) | 0.99 |
